# Prognostic Impact of Chromosome 1q Gain/Amplification in Multiple Myeloma Treated With Daratumumab‐Based Regimens

**DOI:** 10.1111/ejh.70198

**Published:** 2026-04-19

**Authors:** Emiliano Barbieri, Laura Arletti, Micol Quaresima, Elena Rivolti, Luca Braglia, Cecilia Fabiano, Adriana Maria Rita Alfano, Monia Rinaldini, Veronica Bizzarri, Chiara Cattani, Maria Marinelli, Barbara Gamberi

**Affiliations:** ^1^ Hematology Unit, Azienda USL‐IRCCS di Reggio Emilia Reggio Emilia Italy; ^2^ Clinical and Experimental Medicine PhD Program University of Modena and Reggio Emilia Modena Italy; ^3^ Clinical Trials Center, Azienda USL‐IRCCS di Reggio Emilia Reggio Emilia Italy; ^4^ Medical Genetics Unit, Azienda USL‐IRCCS di Reggio Emilia Reggio Emilia Italy

**Keywords:** 1q21, chromosome aberrations, daratumumab, multiple myeloma, prognosis, treatment outcome

## Abstract

Gain/amplification of chromosome arm 1q (+1q) is among the most frequent cytogenetic abnormalities (CAs) in multiple myeloma (MM), and a recognized marker of poor prognosis, now integrated into modern risk stratification systems. The advent of anti‐CD38 monoclonal antibodies, particularly daratumumab, has significantly improved outcomes. However, the prognostic impact of +1q under daratumumab‐based treatments (DBTs) remains uncertain, since pivotal trials rarely reported +1q‐specific outcomes, and available real‐world data are limited and inconsistent. We conducted a single‐center retrospective study of 174 MM patients treated with DBTs between 2018 and 2023 to evaluate the prognostic effect of +1q. By FISH cytogenetic assessment we identified standard‐risk (SR), isolated +1q, +1q plus additional high‐risk CAs (+1q + HiRCAs), and non‐1q HiRCAs groups. The primary endpoint was progression‐free survival (PFS); secondary endpoints were time to next treatment (TTNT) and overall survival (OS). Median age was 72.0 years, median line of DBT administration was 2, and 125 patients (71.8%) received daratumumab‐lenalidomide‐dexamethasone (DaraRd). Cytogenetic data were available for 92 patients (52.9%): 43 SR, 11 non‐1q HiRCAs, 18 isolated +1q and 20 +1q + HiRCAs. After a median follow‐up of 30.7 months, isolated +1q was associated with significantly shorter PFS (HR 4.77, 95% CI: 1.68–13.53) and TTNT (HR 3.83, 95% CI: 1.33–11.09) versus SR, while +1q + HiRCAs had the worst outcomes across all endpoints (PFS HR 7.67; TTNT HR 5.81; OS HR 6.03). Multivariate analysis confirmed isolated +1q as an independent predictor of inferior PFS (HR 4.56, 95% CI: 1.61–12.95) and TTNT (HR 3.64, 95% CI: 1.26–10.55), with +1q + HiRCAs conferring the highest risks on all outcomes (PFS HR 8.36; TTNT HR 6.37; OS HR 6.18). Findings were consistent in the DaraRd subgroup. These findings demonstrate that +1q retains prognostic significance in the era of DBTs. Isolated +1q remains an independent factor of adverse prognosis, further worsened by co‐existing HiRCAs.

## 1 Introduction

Multiple myeloma (MM) is a genomically heterogeneous plasma cell (PC) malignancy in which cytogenetic abnormalities (CAs) strongly influence disease biology and outcomes. Among these, gain or amplification of chromosome arm 1q (+1q) is one of the most frequent with prevalence rising from newly diagnosed (ND) to relapsed/refractory (RR) disease and clonal size expanding with progression, reflecting clonal evolution under selective therapeutic pressure [1, 2].

The adverse prognostic impact of +1q is well established and has been incorporated into contemporary risk models. First recognized in the 2016 International Myeloma Working Group (IMWG) consensus on high‐risk (HiR) cytogenetics [3], +1q was later included in various staging systems, such as the second revision of the ISS [4], and, most recently, integrated into the International Myeloma Society (IMS)/IMWG Consensus Genomic Staging (CGS) on HiRMM [5]. However, in this updated framework, +1q was defined as HiR only when co‐occurring with other adverse CAs, such as t(4;14), t(14;16), t(14;20), or del(1p32). Furthermore, due to heterogeneity in detection thresholds, analytical methods, and clonality assessments, no standardized cut‐off has been defined and no clear distinction was made between gain (gain1q, 3 copies) and amplification (amp1q, > 3 copies).

Despite consensus on its prognostic role, current guidelines [3, 6, 7, 8, 9, 10] remain inconsistent, offering no specific recommendations for +1q, either at diagnosis or relapse, and treatment decisions still rely primarily on prior drug exposure rather than cytogenetic risk.

Historically, trials conducted before the introduction of proteasome inhibitors (PIs) and immunomodulatory drugs (IMiDs) consistently showed poor outcomes for +1q, and these agents did not abrogate its adverse effect, irrespective of autologous stem cell transplantation (ASCT) [2]. In the modern era, no randomized controlled trial (RCT) has been designed specifically for this subgroup, and available data are mostly limited to subgroup analyses or retrospective series [11].

The advent of anti‐CD38 monoclonal antibodies (mAbs), especially daratumumab, has reshaped MM therapy, improving outcomes in both NDMM and RRMM across standard‐risk (SR) and HiR disease [12, 13, 14]. Yet evidence focused on +1q remains limited because pivotal trials seldom reported outcomes for this subgroup and existing data—largely from post hoc analyses or real‐world (RW) series—have yielded conflicting results. Available evidence suggests that daratumumab‐based treatments (DBTs) may mitigate, but not overcome, the adverse effect of +1q, with greatest uncertainty in amp1q or co‐occurring HiRCAs (+1q + HiRCAs) [15].

Adding to this complexity, emerging evidence suggests that resistance mechanisms may differentially affect daratumumab and isatuximab, another anti‐CD38 mAb [2, 11, 15]. While intriguing, these observations remain speculative, require confirmation in comparative studies, and are beyond the scope of this article.

In this context, dedicated analyses are warranted to clarify the prognostic relevance of +1q in the DBT era. Accordingly, we conducted a large single‐center retrospective study evaluating +1q impact in MM patients treated with DBTs.

## 2 Methods

### 2.1 Patients

This single‐center retrospective study included all patients diagnosed with MM according to the IMWG criteria [16] who received at least one cycle of DBT at our institution (Reggio Emilia, Italy) between 1 May 2018 and 31 December 2023. Patients with primary plasma cell leukemia (PCL) or treated with daratumumab monotherapy were excluded. Clinical and laboratory data were extracted from electronic medical records into a dedicated database capturing demographics, disease characteristics, prior therapies, DBT regimen, and follow‐up status. DBT regimens were administered per approved indications and institutional practice. The study protocol was approved by the local Ethics Committee (protocol 2024/0049254) and conducted in accordance with the Declaration of Helsinki. Written informed consent was obtained from all participants.

### 2.2 Cytogenetics

Cytogenetic analyses were performed using fluorescence in situ hybridization (FISH) on CD138+ selected PCs from fresh bone marrow aspirates (magnetic‐bead positive selection; StraightFrom Whole Blood CD138 MicroBeads, Miltenyi Biotec). Probe panels (Cytocell) included t(4;14), t(11;14), t(14;16), del(17p), +1q, and del(1p32). A diagnostic threshold of ≥ 10% of analyzed PCs defined CA positivity. Non‐1q HiRCAs were t(4;14), t(14;16), del(17p), and del(1p32); SR denoted absence of HiRCAs and +1q. Due to incomplete availability of 1q copy number data, +1q was analyzed as an aggregated category without distinguishing between gain 1q and amp 1q. Since baseline FISH is not routinely performed at our center, particularly in older or frail patients, missing data were categorized as not available (NA). In patients with longitudinal FISH, the assessment closest to DBT initiation was used.

### 2.3 Statistical Analysis

Descriptive statistics summarized baseline characteristics, treatments, and outcomes. Disease response, overall response rate (ORR) and progression were assessed according to IMWG criteria [17]. The primary endpoint was progression‐free survival (PFS), defined as the time from DBT initiation to progression or death. Secondary endpoints included time to next treatment (TTNT), defined as the interval between DBT initiation and the start of subsequent line of therapy (LOT) or death, and overall survival (OS), defined as the time from DBT initiation to death from any cause. Patients alive and event‐free were censored at last contact. Survival functions were estimated by Kaplan–Meier (KM) and compared by log‐rank tests. Univariate analyses were conducted for PFS, TTNT and OS across pre‐specified strata (sex, MM subtype, LOT, DBT regimen, cytogenetic status). Numbers‐at‐risk are shown beneath all KM curves. Multivariate analyses were performed using Cox proportional hazards models, with proportional hazard assumption assessed by Schoenfeld residuals. Clinically relevant covariates were pre‐specified and considered for inclusion based on univariate analyses; the final number of variables included in each model was restricted according to the rule of at least 10 events per variable to avoid overfitting. Median follow‐up was estimated by reverse KM. Exact Clopper–Pearson 95% confidence intervals (CIs) were calculated. Analyses were performed using Python 3.11.2.

## 3 Results

### 3.1 Patient Characteristics

A total of 174 patients with MM who received DBTs were included. Baseline characteristics are summarized in Table 1. The median age at DBT initiation was 72.0 years. Renal impairment (creatinine clearance < 60 mL/min) was present in 54 patients (31.0%). Twenty patients (11.5%) exhibited HiR clinical features, including extra‐medullary disease (EMD), secondary PCL, or circulating PCs. Among evaluable patients, 26.5% had ISS stage 3 disease. The median LOT for DBT administration was 2 (range, 1–4), with 80 patients (46.0%) receiving DBTs as first‐ and 73 (42.0%) as second‐line therapy.

**TABLE 1:** Patient characteristics.

Descriptives	Number of patients	% (or range)
Patients enrolled	174	
Sex		
Male	88	50.6
Female	86	49.4
Age		
Median	72.0	48.3–85.9
< 70	72	41.4
≥ 70	102	58.6
Paraprotein (isotype)		
IgG	101	58.0
IgA	40	23.0
IgM	2	1.1
IgD	1	0.6
Light‐chain	28	16.1
Non‐secretory	2	1.1
Light chain restriction		
Kappa	117	67.2
Lambda	57	32.8
Renal insufficiency^a^		
Yes	54	31.0
No	120	69.0
ISS stage		
1	61	35.1
2	36	20.7
3	35	20.1
NA (missing)	42	24.1
High‐risk clinical features		
EMD^b^	12	6.9
Secondary PCL	4	2.3
Circulating PCs < 5%	7	4.0
≥ 1 features	20	11.5
Line of therapy		
1	80	46.0
2	73	42.0
3	14	8.0
4	7	4.0
Median	2	1–4
Previous exposure to PI		
Yes	83	47.7
No	91	52.3
Bortezomib	75	43.1
Carfilzomib	8	4.6
Previous exposure to IMiD		
Yes	73	42.0
No	101	58.0
Thalidomide	44	25.3
Lenalidomide	29	16.7
Previous exposure to anti‐CD38 mAb		
Yes	1	0.6
No	173	99.4
Previous ASCT		
Yes	50	28.7
No	124	71.3
Daratumumab combination		
DaraVTd	21	12.1
DaraRd	125	71.8
DaraVMP	2	1.1
DaraVd	19	10.9
DaraPd	7	4.0
Cytogenetic analysis		
No abnormalities	33	19.0
t(4;14)	13	7.5
t(11;14)	16	9.2
t(14;16)	7	4.0
del(17p)	11	6.3
del(1p32)	7	4.0
+1q	38	21.8
NA (missing)	82	47.1
Cytogenetic status^c^		
Standard‐risk	43	24.7
Non‐1q HiRCAs	11	6.3
Isolated +1q	18	10.3
+1q + HiRCAs	20	11.5
NA	82	47.1
Treatment response^d^		
CR/sCR	73	42.0
VGPR	65	37.4
PR	25	14.4
MinR	4	2.3
SD	2	1.1
PD	3	1.7
NA (missing)	2	1.1
Follow‐up		
Median	30.7	26.3–33.2 (95% CI)

Abbreviations: ASCT, autologous stem cell transplantation; CI, confidence interval; CR, complete response; DaraPd, daratumumab‐pomalidomide‐dexamethasone; DaraRd, daratumumab‐lenalidomide‐dexamethasone; DaraVd, daratumumab‐bortezomib‐dexamethasone; DaraVMP, daratumumab‐bortezomib‐melphalan‐dexamethasone; DaraVTd, daratumumab‐bortezomib‐thalidomide‐dexamethasone; EMD, extra‐medullary disease; HiRCA, high‐risk cytogenetic abnormality; IMiD, immunomodulatory drugs; ISS, International Staging System; mAb, monoclonal antibody; NA, not available; PC, plasma cell; PCL, plasma cell leukemia; PD, progressive disease; PI, proteasome inhibitors; PR, partial response; sCR, stringent complete response; SD, stable disease; VGPR, very good partial response; +1q, gain/amplification of 1q; +1q + HiRCAs, +1q plus ≥ 1 non‐1q HiRCAs.

^a^ Renal insufficiency was defined as creatinine clearance < 60 mL/min, calculated using the CKD‐EPI equation.

^b^ EMD was defined as soft tissue plasmacytomas arising from hematogenous spread with no contact with bony structures, while paraskeletal lesions were excluded.

^c^ Cytogenetic status was defined by FISH analysis. Standard‐risk denoted absence of HiRCAs and +1q. Non‐1q HiRCAs included t(4;14), t(14;16), del(17p), and del(1p32).

^d^ Treatment response was reported in accordance to IMWG criteria [16].

DBT regimens in our cohort were dominated by daratumumab‐lenalidomide‐dexamethasone (DaraRd), administered to 125 patients (71.8%) (Table S1 for baseline characteristics of the DaraRd subgroup). Other regimens included combinations with bortezomib‐thalidomide‐dexamethasone (DaraVTd, *n* = 21), bortezomib‐dexamethasone (DaraVd, *n* = 19), pomalidomide‐dexamethasone (DaraPd, *n* = 7), and bortezomib‐melphalan‐dexamethasone (DaraVMP, *n* = 2) (Table S2 for distribution per LOT).

Cytogenetic analyses were available in 92 patients (52.9%, Table S3). SR was observed in 43 patients (46.7%), +1q in 38 patients (41.3%)—18 as isolated +1q and 20 as +1q + HiRCAs—and non‐1q HiRCAs in 11 patients (12.0%). Baseline characteristics were balanced between patients with and without +1q (Table S4).

### 3.2 Treatment Response

**FIGURE 1:**
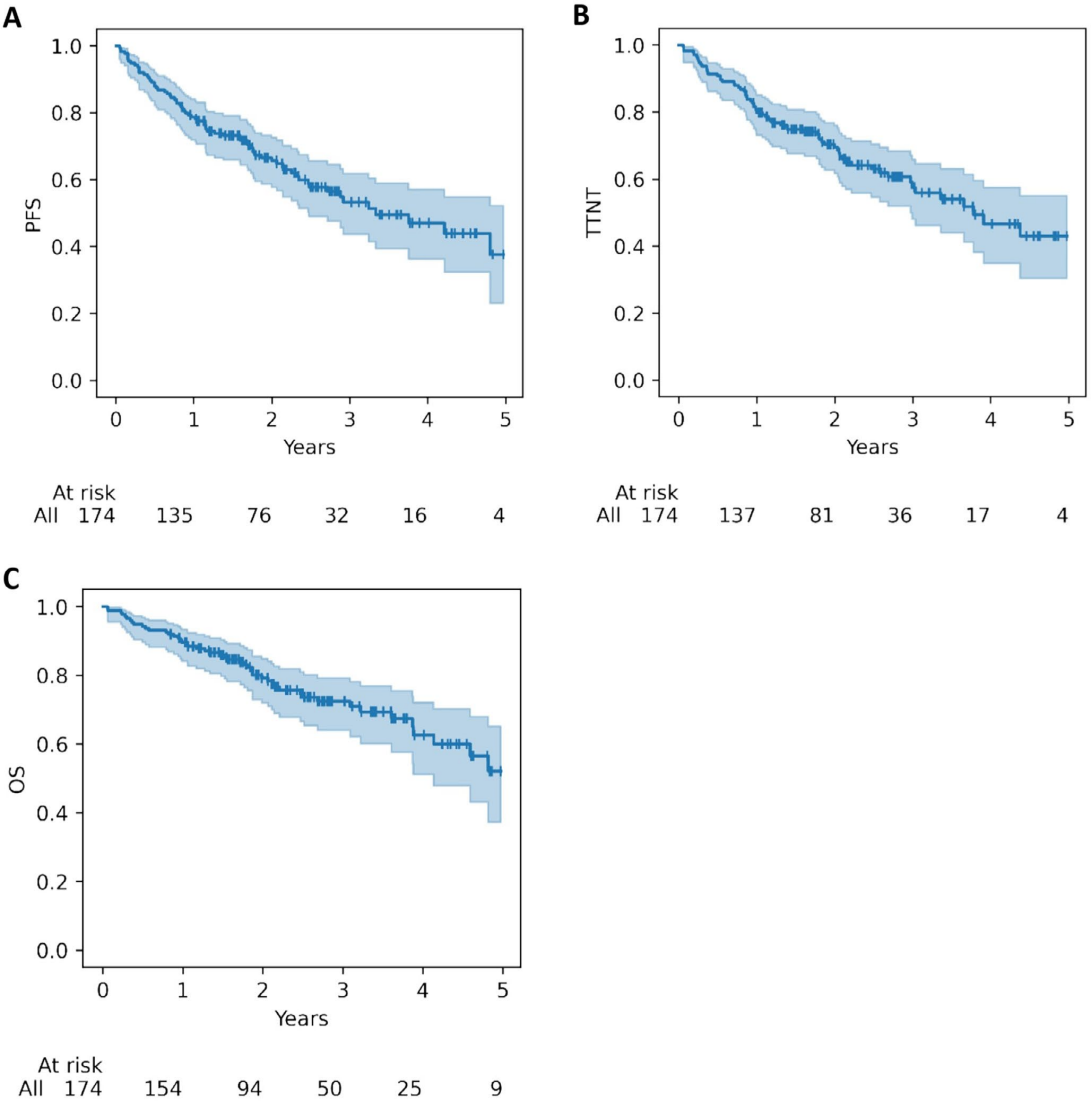
Survival outcomes of the overall cohort. (A) Progression‐free survival. (B) Time to next treatment. (C) Overall survival.

At a median follow‐up of 30.7 months (95% CI: 26.3–33.2), ORR among 172 evaluable patients was 94.8% (95% CI: 90.3–97.6), with 73 patients achieving complete response (CR), 65 very good partial response (VGPR), and 25 partial response (PR). No significant differences were observed between patients with and without +1q (Table S5). Median PFS (mPFS) was 40.0 months (95% CI: 29.8–66.8), median TTNT (mTTNT) 45.4 months [95% CI: 35.6–not‐estimable (NE)], and median OS (mOS) 64.0 months (95% CI: 45.6–NE) (Figure 1). Among 59 patients progressing on DBT, the mPFS of the subsequent LOT was 5.4 months (95% CI: 3.4–9.6).

Results of the DaraRd subgroup are provided in Supporting Information (Figure S1).

### 3.3 Survival Outcomes

#### 3.3.1 Univariate Analysis

**FIGURE 2:**
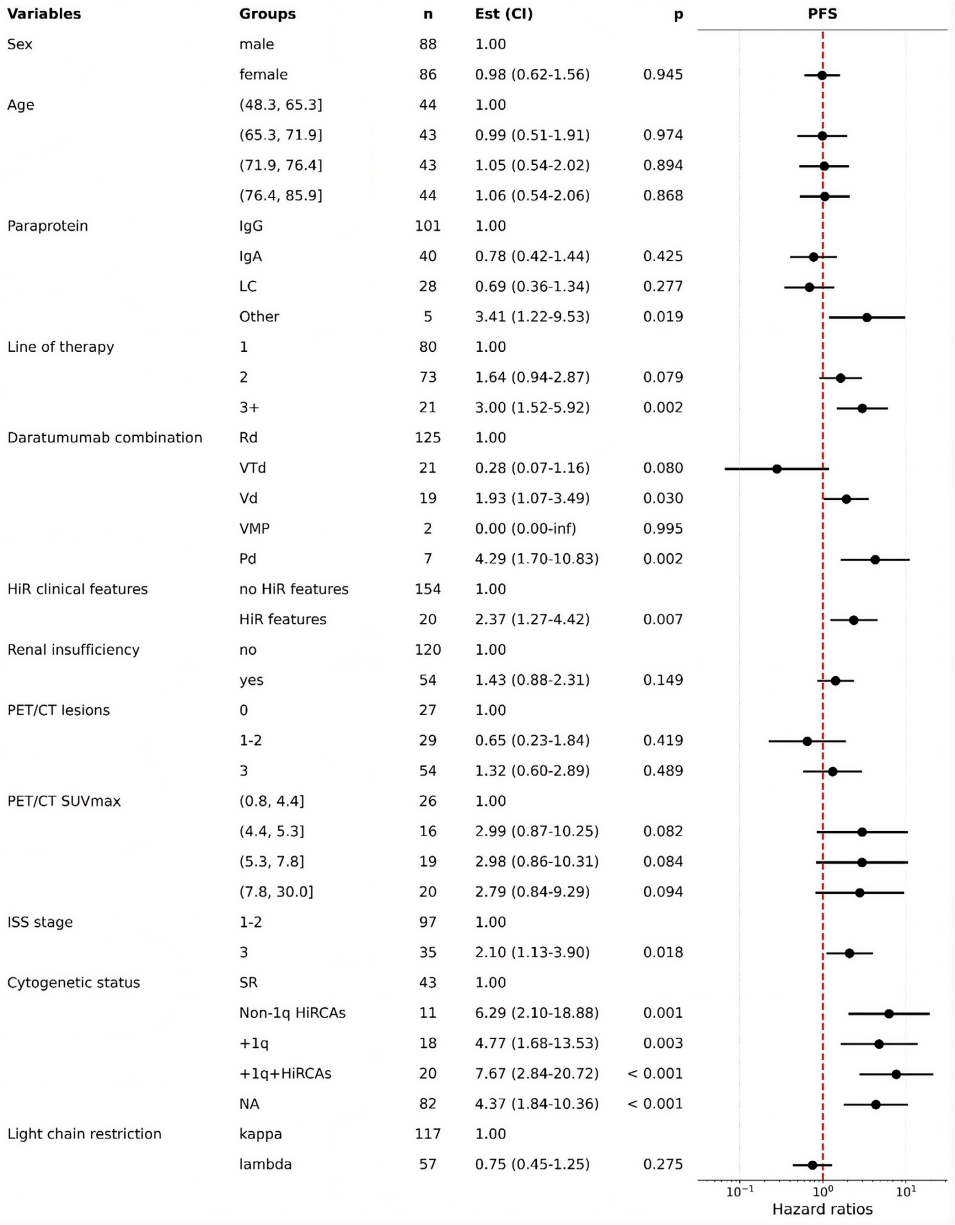
Forest plot of univariate analyses for the primary endpoint PFS. +1q + HiRCAs, +1q plus ≥ 1 non‐1q HiRCAs; +1q, gain/amplification of 1q; CI, 95% confidence of interval; EST, estimates; HiR, high‐risk; HiRCA, high‐risk cytogenetic abnormality; ISS, International Staging System; *n*, number of patients; NA, not available; *p*, *p*‐value; Pd, pomalidomide and dexamethasone; PFS, progression‐free survival; Rd., lenalidomide and dexamethasone; SR, standard‐risk; SUV, standardized uptake value; Vd, bortezomib and dexamethasone; VMP, bortezomib, melphalan and prednisone; VTd, bortezomib, thalidomide and dexamethasone. Other paraprotein isotypes include IgM, IgD and non‐secretory MM. Renal insufficiency was defined as creatinine clearance < 60 mL/min, calculated using the CKD‐EPI equation. HiR clinical features include extra‐medullary disease, secondary plasma cell leukemia, and circulating plasma cells < 5%. Cytogenetic status was defined by FISH analysis. Standard‐risk denoted absence of HiRCAs and +1q. Non‐1q HiRCAs included t(4;14), t(14;16), del(17p), and del(1p32).

**FIGURE 3:**
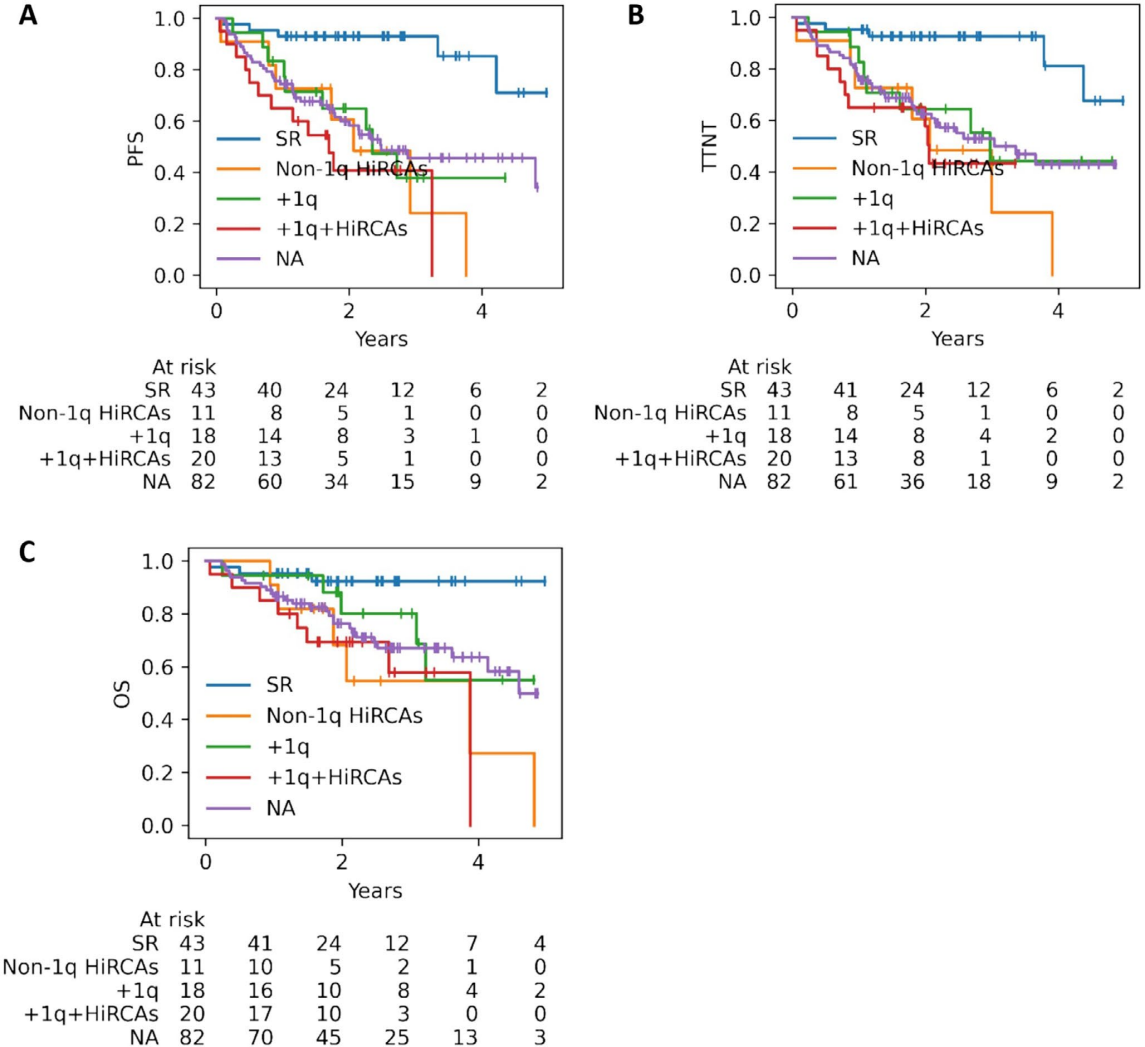
Survival outcomes of the overall cohort stratified by cytogenetic status. (A) Progression‐free survival. (B) Time to next treatment. (C) Overall survival.

Results of univariate analyses for PFS are presented in Figure 2; corresponding analyses for TTNT and OS are provided in Supporting Information, Figures S2 and S3. Cytogenetic risk was strongly associated with outcomes. Compared with SR, isolated +1q was associated with shorter PFS (28.1 vs. 66.8 months, HR 4.77, 95% CI: 1.68–13.53, *p* = 0.003), while the poorest PFS occurred in non‐1q HiRCAs (24.8 months, HR 6.29, 95% CI: 2.10–18.88, *p* = 0.001) and +1q + HiRCAs (20.3 months, HR 7.67, 95% CI: 2.84–20.72, *p* < 0.001). Similar patterns were observed for TTNT, which was shorter in isolated +1q than SR (HR 3.83, 95% CI: 1.33–11.09, *p* = 0.013), but shortest in non‐1q HiRCAs (HR 6.11, 95% CI: 2.04–18.28, *p* = 0.001) and +1q + HiRCAs (HR 5.81, 95% CI: 2.09–16.16, *p* < 0.001). Likewise, OS was inferior in non‐1q HiRCAs (HR 7.05, 95% CI: 1.98–25.10, *p* = 0.003) and +1q + HiRCAs (HR 6.03, 95% CI: 1.78–20.27, *p* = 0.004), while isolated +1q showed only a non‐significant trend toward worse survival (HR 2.69, 95% CI: 0.72–10.01, *p* = 0.141) (Figure 3). To further validate these findings, a sensitivity analysis was performed including only patients with available cytogenetic data (*n* = 92). Results were consistent with the overall cohort, confirming the adverse impact of isolated +1q on PFS and TTNT and a borderline effect on OS, while the poorest outcomes were observed in patients harboring +1q + HiRCAs (Figures S4–S6).

Univariate analyses identified no significant impact of age, sex, or paraprotein isotype on survival. Renal impairment showed a non‐significant trend toward shorter PFS and was associated with shorter TTNT and OS. ISS stage 3 was associated with inferior outcomes compared with stages 1–2, as were HiR clinical features. PET/CT‐defined extent and intensity of skeletal involvement showed no association with outcomes.

Survival declined with later DBT administration, with mPFS decreasing from first‐ to second LOT (NE vs. 34.6 months, HR 1.64, 95% CI: 0.94–2.87, *p* = 0.079) and shortest in subsequent LOTs (13.8 months, HR 3.00, 95% CI: 1.52–5.92, *p* = 0.002). Similar trends were seen for TTNT and OS (Figure S7).

Compared with DaraRd, DaraVTd showed a non‐significant trend toward improved outcomes [mPFS not reached (NR) vs. 45.1 months, HR 0.28, 95% CI: 0.07–1.16, *p* = 0.080; similar trends for TTNT and OS]. Conversely, DaraVd was associated with inferior PFS (20.2 months; HR 1.93, 95% CI: 1.07–3.49, *p* = 0.030) and TTNT, but not OS. DaraPd yielded the poorest outcomes (mPFS 1.8 months, HR 4.29, 95% CI: 1.70–10.83, *p* = 0.002), although this subgroup comprised few, heavily pretreated patients (see Table S2). These trends remained after stratification by LOT (Figures S8–S10).

Interestingly, no post‐DBT salvage regimen significantly influenced survival; PFS curves were comparable across pomalidomide‐based, carfilzomib‐based, and other therapies (Figure S11).

Findings were consistent within the DaraRd subgroup (Supporting Information). Notably, unlike the general cohort, DaraRd effectiveness was consistent across different LOTs, with nearly superimposable PFS, TTNT, and OS curves in first, second, and later LOTs.

#### 3.3.2 Multivariate Analysis

In multivariate models, cytogenetic risk remained the strongest predictor of outcomes. Isolated +1q independently predicted worse PFS (HR 4.56, 95% CI: 1.61–12.95, *p* = 0.004) and TTNT (HR 3.64, 95% CI: 1.26–10.55, *p* = 0.017), with a non‐significant trend for OS (HR 2.62, 95% CI: 0.70–9.79, *p* = 0.151). Non‐1q HiRCAs and +1q + HiRCAs conferred the highest hazards across all endpoints. Later‐line DBT administration was associated with shorter PFS (HR 1.90, 95% CI: 1.11–3.24, *p* = 0.019) and TTNT (HR 2.01, 95% CI: 1.13–3.57, *p* = 0.017), but not OS (HR 1.31, 95% CI: 0.67–2.54, *p* = 0.429) (Table 2).

**TABLE 2:** Multivariate analysis.

Covariate	HR	Lower 95% CI	Upper 95% CI	*p*
PFS				
LOT ≥ 2	1.90	1.11	3.24	0.019
Isolated +1q	4.56	1.61	12.95	0.004
Non‐1q HiRCAs^a^	6.11	2.03	18.35	0.001
+1q + HiRCAs	8.36	3.08	22.70	< 0.001
Cytogenetics NA	4.06	1.71	9.64	0.002
TTNT				
LOT ≥ 2	2.01	1.13	3.57	0.017
Isolated +1q	3.64	1.26	10.55	0.017
Non‐1q HiRCAs	5.90	1.97	17.68	0.002
+1q + HiRCAs	6.37	2.28	17.82	< 0.001
Cytogenetics NA	3.59	1.51	8.55	0.004
OS				
LOT ≥ 2	1.31	0.67	2.54	0.429
Isolated +1q	2.62	0.70	9.79	0.151
Non‐1q HiRCAs	6.91	1.94	24.63	0.003
+1q + HiRCAs	6.18	1.84	20.79	0.003
Cytogenetics NA	3.57	1.24	10.29	0.018

Abbreviations: CI, confidence interval; HiRCA, high‐risk cytogenetic abnormality, HR, hazard ratio; LOT, line of therapy; NA, not available; OS, overall survival; PFS, progression‐free survival; TTNT, time to next treatment; +1q, gain/amplification of 1q; +1q + HiRCAs, +1q plus ≥ 1 non‐1q HiRCAs.

^a^ Non‐1q HiRCAs included t(4;14), t(14;16), del(17p), and del(1p32).

Findings were consistent within the DaraRd subgroup (Supporting Information). Notably, multivariate analysis confirmed that LOT had no independent effect on outcomes in this subgroup (Table S6).

## 4 Discussion

In this single‐center retrospective study, we evaluated outcomes of 174 MM patients treated with DBTs, focusing on the prognostic impact of +1q. To our knowledge, this is among the largest RW analyses of +1q in early LOTs. +1q was consistently associated with inferior outcomes across all survival endpoints, with the most pronounced effect in the presence of concomitant HiRCAs, although isolated +1q also conferred significantly shorter PFS and TTNT. These findings underscore the biological aggressiveness conferred by +1q despite early use of contemporary, highly active DBTs and reinforce +1q as an adverse biomarker in current practice.

Indeed, contemporary risk models recognize +1q as an adverse prognostic factor [2, 11, 15], and the novel IMS/IMWG CGS incorporated it into the HiRMM classification [5], albeit only when co‐occurring with other HiRCAs such as t(4;14), t(14;16), t(14;20), or del(1p32). Importantly, the majority of datasets and clinical studies underpinning the current consensus framework predate widespread daratumumab use, potentially limiting applicability to contemporary DBT regimens, emphasizing the need for further evaluation of +1q under modern treatment backbones.

Notably, DBTs have demonstrated substantial efficacy across NDMM and RRMM, with benefits extending to both SR and HiR disease [12, 13, 14]. However, evidence specific to +1q patients remains limited as most pivotal RCTs did not report dedicated +1q outcomes. Indeed, CASSIOPEIA [18, 19], ALCYONE [20, 21], POLLUX [22, 23], CASTOR [24, 25], CANDOR [26, 27], APOLLO [28, 29] and, more recently, PERSEUS [30] did not provide +1q‐focused results. By contrast, MAIA [31, 32] provided a post hoc subgroup analysis that specifically included +1q patients [33, 34]. In this study on transplant‐ineligible (TI) NDMM patients, adding daratumumab to Rd. appeared to attenuate the adverse impact of +1q (mPFS 53.2 vs. 32.3 months, HR 0.63, 95% CI: 0.46–0.88), yielding PFS estimates that fell between SR and non‐1q HiRCAs. However, this mitigation was less consistent in amp1q and absent in +1q + HiRCA patients. Similarly, the phase II GRIFFIN [35, 36] and MASTER [37, 38] trials in transplant‐eligible (TE) NDMM patients, and the phase III CEPHEUS trial [39, 40] in TI or transplant‐deferred NDMM patients, showed improved outcomes with DBTs in isolated +1q, but not in +1q + HiRCAs, even with quadruplet therapies.

Consistent with these findings, our study confirms that +1q + HiRCAs confer the poorest prognosis, even in a RW population predominantly treated with DaraRd. However, it also suggests that even isolated +1q may be associated with inferior survival. Notably, although direct comparison with MAIA is not appropriate, DaraRd in our cohort performed considerably worse than expected in isolated +1q (mPFS vs. SR patients: 28.2 vs. 66.7 months; HR 8.26, 95% CI: 2.14–31.87), suggesting attenuated benefit in routine practice.

In recent years, several studies have sought to replicate the benefits of DBTs in RW settings and clarify their impact in +1q patients, yet findings remain conflicting [15]. Mohan et al. [41] first reported adverse outcomes associated with +1q in study of 81 heavily pretreated RRMM patients receiving DBTs, showing significantly shorter mPFS (0.5 vs. 2.1 years, *p* < 0.001) and mOS (0.9 years vs. NR, *p* = 0.002). Barbieri et al. [42] reported markedly reduced PFS in amp1q patients (3.0 months vs. NR) within a small cohort of 48 RRMM patients treated with DaraRd or DaraVd. Likewise, in a large RW analysis of 771 RRMM patients from the Czech registry, Stork et al. [43] found no significant PFS benefit for +1q patients with adjunctive daratumumab to Rd. (13.8 vs. 10.2 months, HR 1.33, 95% CI: 0.92–1.91, *p* = 0.129), in contrast to the clear advantage seen in the overall cohort (HR 1.81, 95% CI: 1.43–2.29, *p* < 0.001). More recently, Lim et al. [44] reported significantly shorter PFS for +1q patients receiving DBTs (9.2 vs. 22.1 months, HR 1.60, 95% CI: 1.3–2.1, *p* < 0.001) in a cohort of 536 patients from the Australia–New Zealand and Asia–Pacific registries, association that persisted after multivariate adjustment (HR 1.38, 95% CI: 1.04–1.84, *p* = 0.027). Interestingly, subgroup analyses showed no difference in patients already harboring other HiRCAs, whereas isolated +1q was associated with worse survival than SR (HR 1.80, 95% CI: 1.3–2.4, *p* < 0.001). However, more than 30% of patients were treated beyond second LOT, and DaraRd was underrepresented in favor of DaraVd or daratumumab monotherapy. Similarly, Morabito et al. [45] reported shorter PFS and OS associated with +1q among 635 RRMM patients treated with daratumumab‐, elotuzumab‐, or carfilzomib‐based triplets. Both gain1q and amp1q independently predicted inferior outcomes in multivariate analysis, and concomitant HiRCAs further worsened prognosis. Noteworthy, survivals did not differ by treatment backbone, except for reduced PFS with elotuzumab. In NDMM, Hu et al. [46] also described lack of benefit from DBTs in a small single‐center series of 34 + 1q patients.

By contrast, some RW studies have also reported favorable outcomes. Szabo et al. [47] found isolated +1q outcomes comparable to SR patients (mTTNT 9.8 vs. 11.7 month, HR 1.08, 95% CI: 0.78–1.49, *p* = 0.650) in a cohort of 635 heavily pretreated RRMM patients from the Danish registry, whereas +1q + HiRCAs showed the worst prognosis (HR 1.68, 95% CI: 1.19–2.36, *p* = 0.003), consistent with MAIA findings. Notably, daratumumab–IMiD regimens were significantly more effective than daratumumab–PI combinations or monotherapy. Parrondo et al. [48] similarly suggested that DBTs may mitigate +1q‐associated risk in a series of 232 RRMM patients, where no survival differences were observed between patients with and without +1q (mPFS 24.5 vs. 23.5 months, *p* = 0.392), isolated +1q and SR (*p* = 0.640), or +1q + HiRCAs and non‐1q HiRCAs (*p* = 0.507). However, a few factors may have attenuated the impact of +1q, including predominance of gain1q (54/57 patients), lower treatment burden (second‐line daratumumab 45.6% vs. 29.1%), and more frequent daratumumab‐IMiD combinations used (75.4% vs. 61.7%) in +1q subgroup. Benda et al. [49] also reported favorable outcomes with DBT regimens in a series of 74 RRMM patients. Isolated amp1q had no effect on survival, whereas +1q + HiRCAs was associated with shorter PFS (6.2 vs. 17.3 months, *p* = 0.044) and OS (42.0 vs. 67.0 months for SR patients, *p* = 0.035), suggesting DBTs may overcome the impact of isolated +1q but not +1q + HiRCAs. In NDMM, Kikuchi et al. [50] found no detrimental effect of +1q in a small cohort of 39 DaraRd‐treated patients, with comparable 1‐ and 3‐year PFS to those without +1q (*p* = 0.77).

Overall, our study reinforces evidence from both randomized and RW cohorts indicating that DBTs do not seem to overcome the poor prognosis of +1q + HiRCAs. Furthermore, our findings support the view that even isolated +1q may confer inferior outcomes, albeit with a smaller effect size. Given the heterogeneity across studies and the absence of standardized definitions for +1q, we endorse the IMS/IMWG recommendation for mandatory reporting of analytic methods and thresholds in future MM studies to better define the role of +1q [5].

Our study has several limitations. First, despite the cohort size and focus on +1q, cytogenetic data were missing for a substantial subset, reducing power for subgroup analyses. Moreover, as baseline FISH is not routinely performed at our center in older or frail patients, the group without cytogenetic data (categorized as NA) likely reflects a clinically distinct population; however, findings related to this subgroup should be interpreted with caution. Second, younger, TE patients treated with daratumumab‐based quadruplets were underrepresented and had short follow‐up, limiting generalizability to this subgroup. Third, although follow‐up was comparable or longer than most published series, it remains insufficient for mature OS estimates. Finally, sample size and missing data precluded robust comparison of gain1q versus amp1q, limiting conclusions on copy number‐dependent effects.

## 5 Conclusion

This RW study, predominantly composed of patients treated with DBT triplets—mainly DaraRd—in early LOTs, confirms the HiR profile of +1q when co‐occurring with other HiRCAs, as recognized in the IMS/IMWG consensus, and further suggests that even isolated +1q may carry adverse prognostic significance in RW practice. Whether emerging daratumumab‐based quadruplets, absent from our cohort but highly promising in RCTs, can improve outcomes in +1q‐defined subgroups remains to be determined. Comparative evaluation of isatuximab‐based regimens is also warranted, given encouraging evidence in +1q patients. Our findings highlight the need for risk‐adapted strategies and early referral of these HiR patients to novel therapies, including bispecific antibodies and CAR‐T cells, ideally within RCTs. Finally, standardized definitions and reporting of +1q, including copy‐number status, will be essential to refine risk stratification and clarify its role in the modern therapeutic era.

## Funding

The authors have nothing to report.

## Ethics Statement

The study was conducted in accordance with the Declaration of Helsinki and approved by the local Ethics Committee (approval number 2024/0049254).

## Consent

All patients provided written informed consent; for deceased individuals, the use of anonymized data was approved by the local Ethics Committee in accordance with national regulations.

## Conflicts of Interest

Barbara Gamberi has received consultancy fees and honoraria from Takeda, Sanofi, Johnson & Johnson, Pfizer, Menarini Stemline, GlaxoSmithKline, and Amgen. The other authors declare no conflicts of interest.

## Supporting information


**Table S1:** Patient characteristics—DaraRd subgroup.
**Table S2:** Number of patients per treatment line and daratumumab combination.
**Table S3:** Patient characteristics—subgroup of patient with available cytogenetic data.
**Table S4:** Comparison of baseline characteristics between patients with and without +1q.
**Table S5:** ORR and CR rate per cytogenetic subgroup.
**Table S6:** Multivariate analysis—DaraRd subgroup.
**Figure S1:** Survival outcomes of the DaraRd subgroup. (A) Progression‐free survival. (B) Time to next treatment. (C) Overall survival.
**Figure S2:** Forest plot of univariate analyses for the secondary outcome TTNT.
**Figure S3:** Forest plot of univariate analyses for the secondary outcome OS.
**Figure S4:** Univariate analysis of PFS in patients with available cytogenetic data.
**Figure S5:** Univariate analysis of TTNT in patients with available cytogenetic data.
**Figure S6:** Univariate analysis of OS in patients with available cytogenetic data.
**Figure S7:** Survival outcomes of the overall cohort stratified by line of treatment. (A) Progression‐free survival. (B) Time to next treatment. (C) Overall survival.
**Figure S8:** Survival outcomes of the overall cohort stratified by treatment scheme. (A) Progression‐free survival. (B) Time to next treatment. (C) Overall survival.
**Figure S9:** Survival outcomes of NDMM patients (i.e., first‐line treatment) stratified by treatment scheme. (A) Progression‐free survival. (B) Time to next treatment. (C) Overall survival.
**Figure S10:** Survival outcomes of RRMM patients (i.e., ≥ 2 lines of treatment) stratified by treatment scheme. (A) Progression‐free survival. (B) Time to next treatment. (C) Overall survival.
**Figure S11:** Progression‐free survival of patients relapsed/refractory to daratumumab‐based treatments stratified by salvage treatment scheme.
**Figure S12:** Survival outcomes of the DaraRd subgroup stratified by cytogenetic status. (A) Progression‐free survival. (B) Time to next treatment. (C) Overall survival.
**Figure S13:** Survival outcomes of the DaraRd subgroup stratified by line of treatment. (A) Progression‐free survival. (B) Time to next treatment. (C) Overall survival.

## Data Availability

The data that support the findings of this study are available from the corresponding author upon reasonable request.
