# Supplementary material for: Prognostic Impact of Chromosome 1q Gain/Amplification in Multiple Myeloma Treated With Daratumumab‐Based Regimens
Source: Eur J Haematol. 2026 Apr 19;117(2):409–21. doi: 10.1111/ejh.70198 (PMC13326790; doi:10.1111/ejh.70198)
Supplement: Supplementary file 1 — Table S1: Patient characteristics—DaraRd subgroup. Table S2: Number of patients per treatment line and daratumumab combination. Table S3: Patient characteristics—subgroup of patient with available cytogenetic data. Table S4: Comparison of baseline characteristics between patients with and without +1q. Table S5: ORR and CR rate per cytogenetic subgroup. Table S6: Multivariate analysis—DaraRd subgroup. Figure S1: Survival outcomes of the DaraRd subgroup. (A) Progression‐free survival. (B) Time to next treatment. (C) Overall survival. Figure S2: Forest plot of univariate analyses for the secondary outcome TTNT. Figure S3: Forest plot of univariate analyses for the secondary outcome OS. Figure S4: Univariate analysis of PFS in patients with available cytogenetic data. Figure S5: Univariate analysis of TTNT in patients with available cytogenetic data. Figure S6: Univariate analysis of OS in patients with available cytogenetic data. Figure S7: Survival outcomes of the overall cohort stratified by line of treatment. (A) Progression‐free survival. (B) Time to next treatment. (C) Overall survival. Figure S8: Survival outcomes of the overall cohort stratified by treatment scheme. (A) Progression‐free survival. (B) Time to next treatment. (C) Overall survival. Figure S9: Survival outcomes of NDMM patients (i.e., first‐line treatment) stratified by treatment scheme. (A) Progression‐free survival. (B) Time to next treatment. (C) Overall survival. Figure S10: Survival outcomes of RRMM patients (i.e., ≥ 2 lines of treatment) stratified by treatment scheme. (A) Progression‐free survival. (B) Time to next treatment. (C) Overall survival. Figure S11: Progression‐free survival of patients relapsed/refractory to daratumumab‐based treatments stratified by salvage treatment scheme. Figure S12: Survival outcomes of the DaraRd subgroup stratified by cytogenetic status. (A) Progression‐free survival. (B) Time to next treatment. (C) Overall survival. Figure S13: Survival outcomes of the Dara [file EJH-117-409-s001.docx]

SUPPLEMENTARY MATERIALS

RESULTS

TREATMENT RESPONSE – DARARD SUBGROUP

For the DaraRd subgroup (n=125), ORR was 96.8%, with CR achieved in 54 patients (43.2%), VGPR in 49 (39.2%), and PR in 18 (14.4%). The mPFS was 45.1 months (95% CI 29.6–NE), while mTTNT and mOS were NE due to the low number of events at data cutoff (Figure S1).

SURVIVAL OUTCOMES

UNIVARIATE ANALYSIS – DARARD SUBGROUP

Cytogenetic risk was strongly associated with outcomes in the DaraRd cohort. Patients with isolated +1q had significantly shorter PFS compared to SR patients (28.2 vs 66.7 months, HR 8.26, 95% CI 2.14–31.87, p=0.002) and TTNT (HR 5.84, 95% CI 1.50–22.75, p=0.010), with a non-significant trend for OS (HR 3.75, 95% CI 0.69–20.49, p=0.127). The worst outcomes were seen for patients with non-1q HiRCAs (mPFS 24.7 months, HR 10.24, 95% CI 2.40–43.69, p=0.002; mTTNT HR 8.99, 95% CI 2.13–37.93, p=0.003; mOS HR 10.73, 95% CI 1.95–59.12, p=0.006) and +1q+HiRCAs (mPFS 20.3 months, HR 11.30, 95% CI 2.99–42.78, p<0.001; mTTNT HR 7.09, 95% CI 1.81–27.73, p=0.005; mOS HR 7.59, 95% CI 1.51–30.02, p=0.014) (Figure S12).

In contrast to the overall cohort, DaraRd demonstrated consistent effectiveness across different LOTs. PFS curves were nearly superimposable whether DaraRd was administered in first, second, or later lines (mPFS: NE vs 45.1 vs 40.0 months; HR 1.17, 95% CI 0.64–2.16, p=0.610; HR 1.46, 95% CI 0.57–3.71, p=0.431), with similar patterns observed for TTNT and OS (Figure S13).

Again, factors such as age, sex and type of paraprotein did not influence outcomes.

MULTIVARIATE ANALYSIS – DARARD SUBGROUP

Isolated +1q was independently associated with inferior PFS (HR 8.20, 95% CI 2.12–31.66, p=0.002) and TTNT (HR 5.77, 95% CI 1.48–22.46, p=0.012), with a non-significant trend for OS (HR 3.75, 95% CI 0.69–20.50, p=0.127). Similar to the overall cohort, non-1q HiRCAs and +1q+HiRCAs retained the strongest prognostic impact for all outcomes. Multivariate analysis confirmed that treatment line had no independent effect on outcomes in this subgroup (*Table S6*).

SUPPLEMENTARY TABLES

| Table S1. Patient characteristics - DaraRd subgroup. | | | |
| --- | --- | --- | --- |
| Descriptives | | N. of patients | % (or range) |
| Patients enrolled |  | 125 |  |
| Sex | Male | 61 | 48.8 |
|  | Female | 64 | 51.2 |
| Age | Median  <70  ≥70 | 72.7  41  84 | 48.3 – 85.9 (range)  32.8  67.2 |
| Paraprotein (isotype) | IgG | 71 | 56.8 |
|  | IgA  IgM  IgD  Light-chain  Non-secretory | 27  2  0  24  1 | 21.6  1.6  0.0  19.2  0.8 |
| Light chain restriction | Kappa | 81 | 64.8 |
|  | Lambda | 44 | 35.2 |
| Renal insufficiency† | Yes  No | 44  81 | 35.2  64.8 |
| ISS stage | 1  2  3  NA (missing) | 43  25  28  29 | 34.4  20.0  22.4  23.2 |
| High-risk clinical features | EMD‡  Secondary PCL  Circulating PCs < 5%  ≥ 1 features | 8  3  6  15 | 6.4  2.4  4.8  12.0 |
| Line of therapy | 1 | 57 | 45.6 |
|  | 2 | 58 | 46.4 |
|  | 3 | 7 | 5.6 |
|  | 4  Median | 3  2 | 2.4  1 – 4 (range) |
| Previous exposure to PI | Yes  No  Bortezomib  Carfilzomib | 65  60  63  2 | 52.0  48.0  50.4  1.6 |
| Previous exposure to IMID | Yes  No  Thalidomide  Lenalidomide | 43  82  37  6 | 34.4  65.6  29.6  4.8 |
| Previous exposure to anti-CD38 mAb | Yes  No | 0  125 | 0.0  100.0 |
| Previous ASCT | Yes  No | 35  90 | 28.0  72.0 |
| Cytogenetic analysis | No abnormalities  t(4;14)  t(11;14)  t(14;16)  del(17p)  del(1p32)  +1q  NA (missing) | 19  10  10  4  7  4  28  63 | 15.2  8.0  8.0  3.2  5.6  3.2  22.4  50.4 |
| Cytogenetic status§ | Standard-risk  Non-1q HiRCAs | 27  7 | 21.6  5.6 |
|  | Isolated +1q  +1q+HiRCAs  NA | 14  14  63 | 11.2  11.2  50.4 |
| Treatment response¶ | CR/sCR | 54 | 43.2 |
|  | VGPR | 49 | 39.2 |
|  | PR | 18 | 14.4 |
|  | MinR | 2 | 1.6 |
|  | SD | 1 | 0.8 |
|  | PD | 1 | 0.8 |
|  | NA (missing) | 0 | 0.0 |
| Follow-up | Median | 32.9 | 29.2 – 34.3 (95% CI) |
| ASCT= autologous stem cell transplantation; CI= confidence interval; CR= complete response; DaraRd= daratumumab-lenalidomide-dexamethasone; EMD= extra-medullary disease; HiRCA= high-risk cytogenetic abnormality; IMiD= immunomodulatory drugs; ISS= International Staging System; mAb= monoclonal antibody; NA= not available; PC= plasma cell; PCL= plasma cell leukemia; PD= progressive disease; PI= proteasome inhibitors; PR= partial response; sCR= stringent complete response; SD= stable disease; VGPR= very good partial response; +1q= gain/amplification of 1q; +1q+HiRCAs= +1q plus ≥ 1 non-1q HiRCAs.  †Renal insufficiency was defined as creatinine clearance <60 mL/min, calculated using the CKD-EPI equation.  ‡EMD was defined as soft tissue plasmacytomas arising from hematogenous spread with no contact with bony structures, while paraskeletal lesions were excluded.  §Cytogenetic status was defined by FISH analysis. Standard-risk denoted absence of HiRCAs and +1q. Non-1q HiRCAs included t(4;14), t(14;16), del(17p), and del(1p32).  ¶Treatment response was reported in accordance to IMWG criteria [16]. | | | |

| Table S2. Number of patients per treatment line and daratumumab combination. | | | | | |
| --- | --- | --- | --- | --- | --- |
|  | 1 | 2 | 3 | 4 | Overall |
| DaraRd | 57 | 58 | 7 | 3 | 125 |
| DaraVTd | 21 | 0 | 0 | 0 | 21 |
| DaraVd | 0 | 14 | 4 | 1 | 19 |
| DaraPd | 0 | 1 | 3 | 3 | 7 |
| DaraVMP | 2 | 0 | 0 | 0 | 2 |
| Overall | 80 | 73 | 14 | 7 | 174 |
| DaraPd= daratumumab-pomalidomide-dexamethasone; DaraRd= daratumumab-lenalidomide-dexamethasone; DaraVd= daratumumab-bortezomib-dexamethasone; DaraVMP= daratumumab-bortezomib-melphalan-dexamethasone; DaraVTd= daratumumab-bortezomib-thalidomide-dexamethasone. | | | | | |

| Table S3. Patient characteristics – subgroup of patient with available cytogenetic data | | | |
| --- | --- | --- | --- |
| Descriptives | | N. of patients | % (or range) |
| Patients enrolled |  | 92 |  |
| Sex | Male | 49 | 53.3 |
|  | Female | 43 | 46.7 |
| Age | Median  <70  ≥70 | 67.8  53  39 | 48.3 – 85.9  57.6  42.4 |
| Paraprotein (isotype) | IgG | 51 | 55.4 |
|  | IgA  IgM  IgD  Light-chain  Non-secretory | 25  0  1  14  1 | 27.2  0.0  1.1  15.2  1.1 |
| Light chain restriction | Kappa | 73 | 79.3 |
|  | Lambda | 19 | 20.7 |
| Renal insufficiency† | Yes  No | 28  64 | 30.4  69.6 |
| ISS stage | 1  2  3  NA (missing) | 34  20  22  16 | 37.0  21.7  23.9  17.4 |
| High-risk clinical features | EMD‡  Secondary PCL  Circulating PCs < 5%  ≥ 1 features | 7  1  3  9 | 7.6  1.1  3.3  9.8 |
| Line of therapy | 1 | 50 | 54.4 |
|  | 2 | 31 | 33.7 |
|  | 3 | 7 | 7.6 |
|  | 4  Median | 4  1 | 4.3  1 – 4 |
| Previous exposure to PI | Yes  No  Bortezomib  Carfilzomib | 36  56  34  6 | 39.1  60.9  37.0  6.5 |
| Previous exposure to IMiD | Yes  No  Thalidomide  Lenalidomide | 36  56  29  11 | 39.1  60.9  31.5  12.0 |
| Previous exposure to anti-CD38 mAb | Yes  No | 1  91 | 1.1  98.9 |
| Previous ASCT | Yes  No | 69  23 | 75.0  25.0 |
| Daratumumab combination | DaraVTd  DaraRd  DaraVMP  DaraVd  DaraPd | 19  62  1  5  5 | 20.7  67.4  1.1  5.4  5.4 |
| Cytogenetic analysis | No abnormalities  t(4;14)  t(11;14)  t(14;16)  del(17p)  del(1p32)  +1q | 33  13  16  7  11  7  38 | 19.0  7.5  9.2  4.0  6.3  4.0  21.8 |
| Cytogenetic status§ | Standard-risk  Non-1q HiRCAs | 43  11 | 24.7  6.3 |
|  | Isolated +1q  +1q+HiRCAs | 18  20 | 10.3  11.5 |
| Treatment response¶ | CR/sCR | 41 | 44.5 |
|  | VGPR | 35 | 38.0 |
|  | PR | 11 | 12.0 |
|  | PD | 3 | 3.3 |
|  | NA (missing) | 2 | 2.2 |
| Follow-up | Median | 30.0 | 24.7 – 33.2 (95% CI) |
| ASCT= autologous stem cell transplantation; CI= confidence interval; CR= complete response; DaraPd= daratumumab-pomalidomide-dexamethasone; DaraRd= daratumumab-lenalidomide-dexamethasone; DaraVd= daratumumab-bortezomib-dexamethasone; DaraVMP= daratumumab-bortezomib-melphalan-dexamethasone; DaraVTd= daratumumab-bortezomib-thalidomide-dexamethasone; EMD= extra-medullary disease; HiRCA= high-risk cytogenetic abnormality; IMiD= immunomodulatory drugs; ISS= International Staging System; mAb= monoclonal antibody; NA= not available; PC= plasma cell; PCL= plasma cell leukemia; PD= progressive disease; PI= proteasome inhibitors; PR= partial response; sCR= stringent complete response; SD= stable disease; VGPR= very good partial response; +1q= gain/amplification of 1q. +1q+HiRCAs= +1q plus ≥ 1 non-1q HiRCAs.  †Renal insufficiency was defined as creatinine clearance <60 mL/min, calculated using the CKD-EPI equation.  ‡EMD was defined as soft tissue plasmacytomas arising from hematogenous spread with no contact with bony structures, while paraskeletal lesions were excluded.  §Cytogenetic status was defined by FISH analysis. Standard-risk denoted absence of HiRCAs and +1q. Non-1q HiRCAs included t(4;14), t(14;16), del(17p), and del(1p32).  ¶Treatment response was reported in accordance to IMWG criteria [16]. | | | |

| Table S4 Comparison of baseline characteristics between patients with and without +1q. | | | | | |
| --- | --- | --- | --- | --- | --- |
|  |  | Overall | +1q | No +1q | p-value |
| n |  | 92 | 38 | 54 |  |
| Age (median) |  | 67.8 | 67.6 | 67.9 | 0.709 |
| Sex | Male | 49 (53.3%) | 15 (39.5%) | 34 (63.0%) | 0.044 |
|  | Female | 43 (46.7%) | 23 (60.5%) | 20 (37.0%) |  |
| Paraprotein | IgG | 51 (55.4%) | 20 (52.6%) | 31 (57.4%) | 0.506 |
|  | IgA | 25 (27.2%) | 10 (26.3%) | 15 (27.8%) |  |
|  | LC | 14 (15.2%) | 8 (21.1%) | 6 (11.1%) |  |
|  | Other† | 2 (2.2%) | 0 (0.0%) | 2 (3.7%) |  |
| Renal insufficiency‡ | No | 64 (69.6%) | 24 (63.2%) | 40 (74.1%) | 0.373 |
|  | Yes | 28 (30.4%) | 14 (36.8%) | 14 (25.9%) |  |
| ISS stage | 1-2 | 54 (71.1%) | 21 (65.6%) | 33 (75.0%) | 0.526 |
|  | 3 | 22 (28.9%) | 11 (34.4%) | 11 (25.0%) |  |
| HiR clinical features§ | No | 83 (90.2%) | 33 (86.8%) | 50 (92.6%) | 0.480 |
|  | Yes | 9 (9.8%) | 5 (13.2%) | 4 (7.4%) |  |
| Line of therapy | 1 | 50 (54.3%) | 19 (50.0%) | 31 (57.4%) | 0.624 |
|  | ≥2 | 42 (45.7%) | 19 (50.0%) | 23 (42.6%) |  |
| HiR= high-risk; ISS= International Staging System; +1q= gain/amplification of 1q.  †Other paraprotein isotypes include IgM, IgD and non-secretory MM ‡Renal insufficiency was defined as creatinine clearance <60 mL/min, calculated using the CKD-EPI equation  §HiR clinical features include extra-medullary disease, secondary plasma cell leukemia and circulating plasma cells < 5%. | | | | | |

| Table S5. ORR and CR rate per cytogenetic subgroup. | | | | | | | | |
| --- | --- | --- | --- | --- | --- | --- | --- | --- |
|  |  | Missing | Overall | SR | Non-1q HiRCAs† | +1q | +1q+HiRCAs | p-value |
| n |  |  | 92 | 43 | 11 | 18 | 20 |  |
| ORR (%) | No | 2 | 3 (3.3%) | 0 (0.0%) | 1 (9.1%) | 0 (0.0%) | 2 (10.0%) | 0.115 |
|  | Yes |  | 87 (96.7%) | 42 (100.0%) | 10 (90.9%) | 17 (100.0%) | 18 (90.0%) |  |
| CR (%) | No | 0 | 41 (44.6%) | 19 (44.2%) | 6 (54.5%) | 8 (44.4%) | 8 (40.0%) | 0.890 |
|  | Yes |  | 51 (55.4%) | 24 (55.8%) | 5 (45.5%) | 10 (55.6%) | 12 (60.0%) |  |
| CR= complete response; HiRCA= high-risk cytogenetic abnormality; n= number of patients; ORR= overall response rate; SR= standard-risk; +1q= gain/amplification of 1q; +1q+HiRCAs= +1q plus ≥ 1 non-1q HiRCAs.  †Non-1q HiRCAs included t(4;14), t(14;16), del(17p), and del(1p32). | | | | | | | | |

| Table S6. Multivariate analysis - DaraRd subgroup. | | | | |
| --- | --- | --- | --- | --- |
| PFS | | | | |
| Covariate | HR | Lower 95% CI | Upper 95% CI | p-value |
| LOT ≥2 | 1.22 | 0.67 | 2.20 | 0.517 |
| Isolated +1q | 8.20 | 2.12 | 31.66 | 0.002 |
| Non-1q HiRCAs† | 10.10 | 2.37 | 43.09 | 0.002 |
| +1q+HiRCAs | 11.50 | 3.03 | 43.60 | <0.001 |
| Cytogenetics NA | 5.34 | 1.61 | 17.74 | 0.006 |
|  | | | | |
| TTNT | | | | |
| Covariate | HR | Lower 95% CI | Upper 95% CI | p-value |
| LOT ≥2 | 1.37 | 0.73 | 2.58 | 0.325 |
| Isolated +1q | 5.77 | 1.48 | 22.46 | 0.012 |
| Non-1q HiRCAs | 8.77 | 2.08 | 37.04 | 0.003 |
| +1q+HiRCAs | 7.28 | 1.86 | 28.51 | 0.004 |
| Cytogenetics NA | 4.45 | 1.34 | 14.77 | 0.015 |
|  | | | | |
| OS | | | | |
| Covariate | HR | Lower 95% CI | Upper 95% CI | p-value |
| LOT ≥2 | 0.99 | 0.49 | 2.03 | 0.985 |
| Isolated +1q | 3.75 | 0.69 | 20.50 | 0.127 |
| Non-1q HiRCAs | 10.74 | 1.95 | 59.25 | 0.006 |
| +1q+HiRCAs | 7.59 | 1.51 | 38.02 | 0.014 |
| Cytogenetics NA | 5.64 | 1.32 | 24.15 | 0.020 |
| CI= confidence interval; HiRCA= high-risk cytogenetic abnormality, HR= hazard ratio; LOT= line of therapy; NA= not available; OS= overall survival; PFS= progression-free survival; TTNT= time to next treatment; +1q= gain/amplification of 1q; +1q+HiRCAs= +1q plus ≥ 1 non-1q HiRCAs. †Non-1q HiRCAs included t(4;14), t(14;16), del(17p), and del(1p32). | | | | |

SUPPLEMENTARY FIGURES


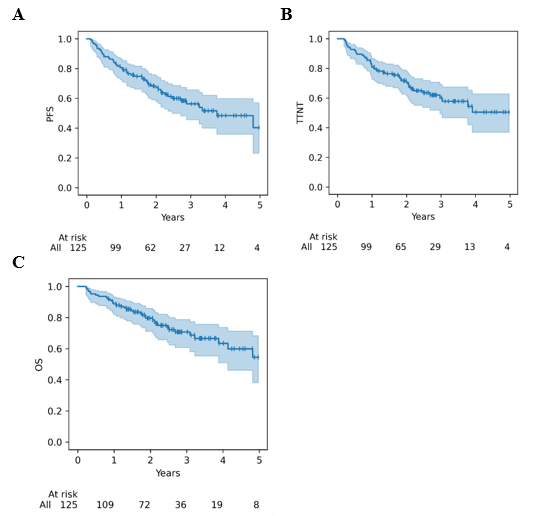


**Figure S1.** Survival outcomes of the DaraRd subgroup. **A.** Progression-Free Survival. **B.** Time to next treatment. **C.** Overall Survival.


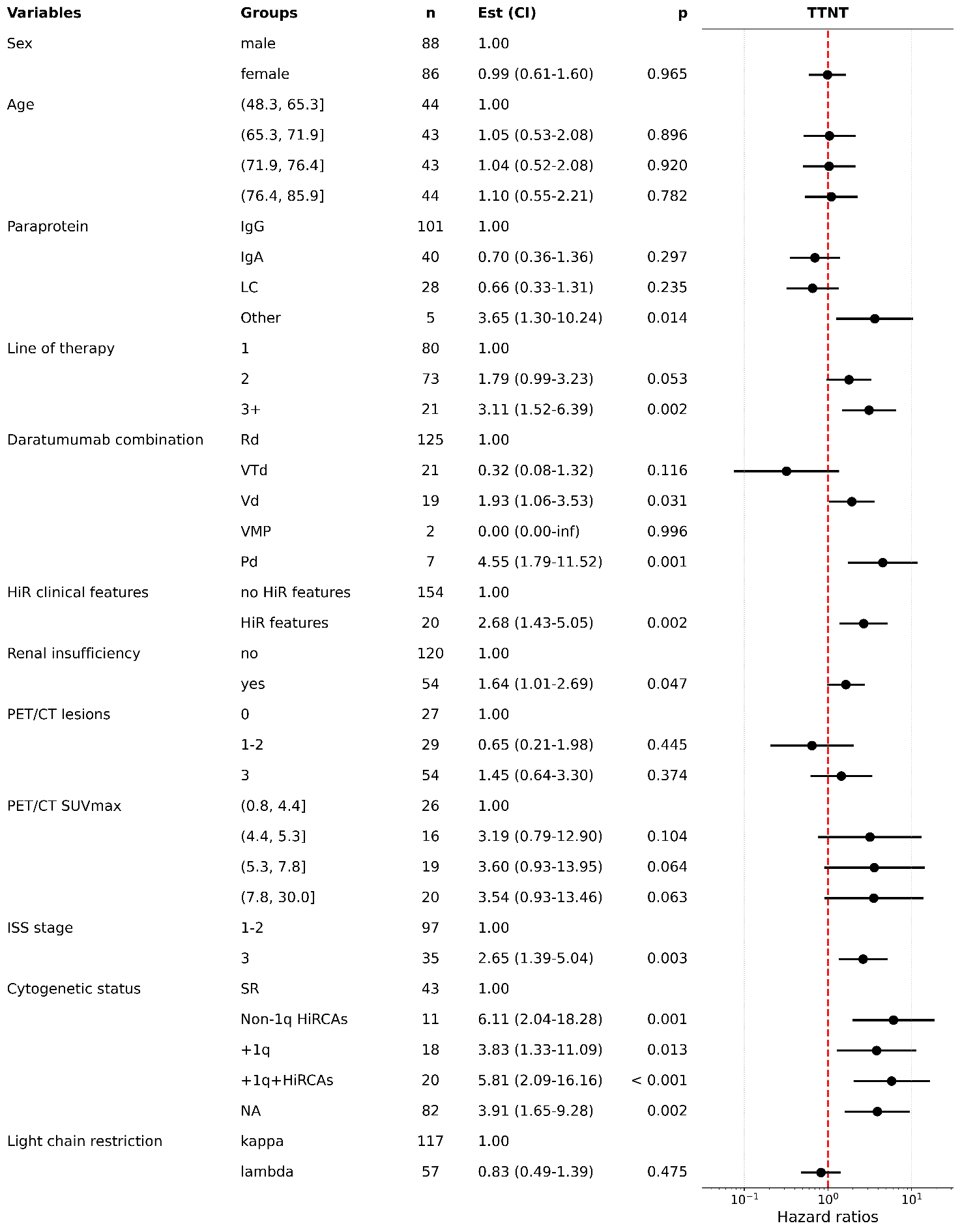


**Figure S2.** Forest plot of univariate analyses for the secondary outcome TTNT.
CI= 95% confidence of interval; EST= estimates; HiR= high-risk; HiRCA= high-risk cytogenetic abnormality; ISS= International Staging System; n= number of patients; NA= not available; p= p-value; Pd= pomalidomide and dexamethasone; Rd= lenalidomide and dexamethasone; SR= standard-risk; SUV= standardized uptake value; TTNT= time to next treatment; Vd= bortezomib and dexamethasone; VMP= bortezomib, melphalan and prednisone; VTd= bortezomib, thalidomide and dexamethasone; +1q= gain/amplification of 1q; +1q+HiRCAs= +1q plus ≥ 1 non-1q HiRCAs.
Other paraprotein isotypes include IgM, IgD and non-secretory MM.
Renal insufficiency was defined as creatinine clearance <60 mL/min, calculated using the CKD-EPI equation.
HiR clinical features include extra-medullary disease, secondary plasma cell leukemia and circulating plasma cells < 5%.
Cytogenetic status was defined by FISH analysis. Standard-risk denoted absence of HiRCAs and +1q. Non-1q HiRCAs included t(4;14), t(14;16), del(17p), and del(1p32).


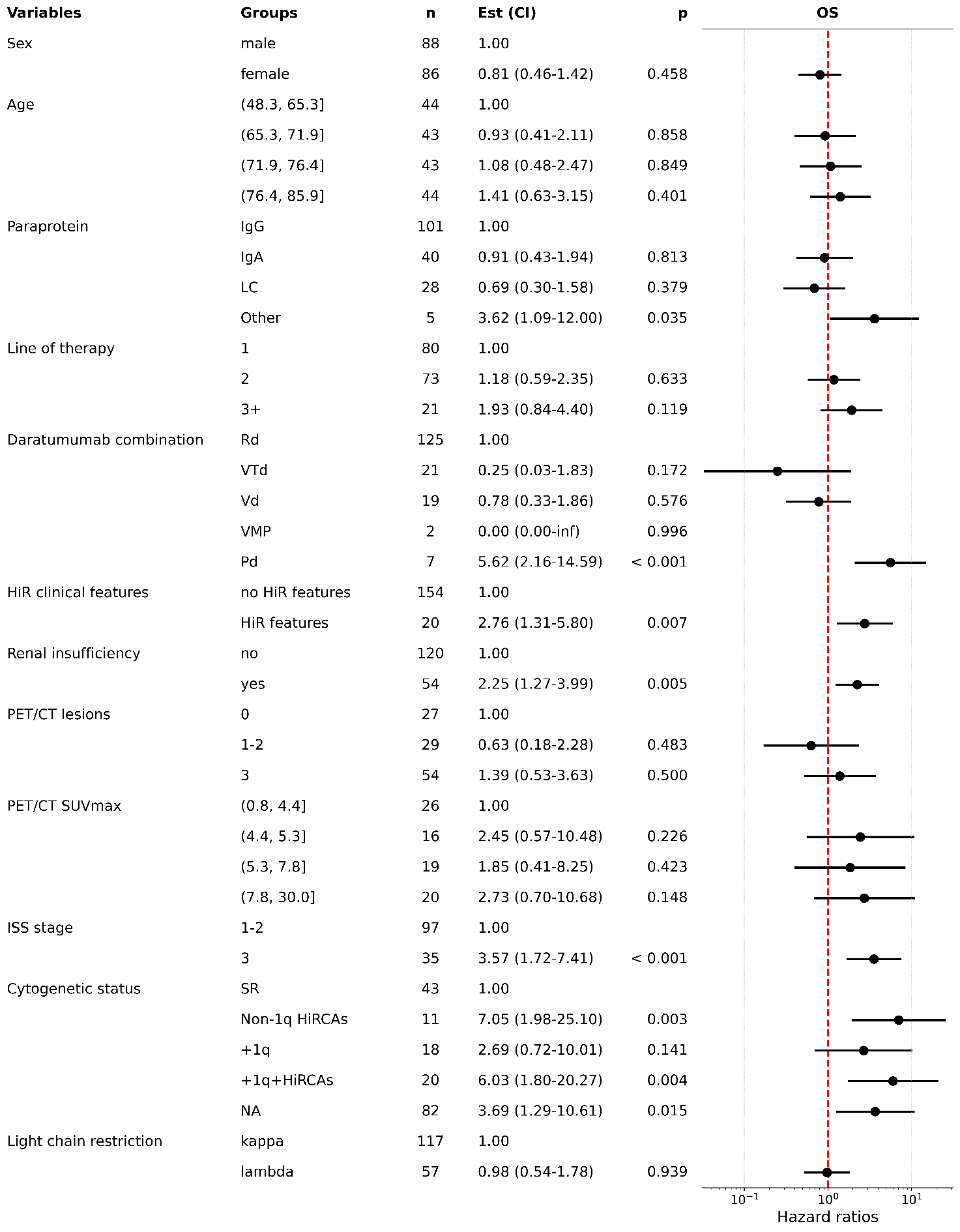


**Figure S3.** Forest plot of univariate analyses for the secondary outcome OS.
CI= 95% confidence of interval; EST= estimates; HiR= high-risk; HiRCA= high-risk cytogenetic abnormality; ISS= International Staging System; n= number of patients; NA= not available; OS= overall survival; p= p-value; Pd= pomalidomide and dexamethasone; Rd= lenalidomide and dexamethasone; SR= standard-risk; SUV= standardized uptake value; Vd= bortezomib and dexamethasone; VMP= bortezomib, melphalan and prednisone; VTd= bortezomib, thalidomide and dexamethasone; +1q= gain/amplification of 1q; +1q+HiRCAs= +1q plus ≥ 1 non-1q HiRCAs.
Other paraprotein isotypes include IgM, IgD and non-secretory MM.
Renal insufficiency was defined as creatinine clearance <60 mL/min, calculated using the CKD-EPI equation.
HiR clinical features include extra-medullary disease, secondary plasma cell leukemia and circulating plasma cells < 5%.
Cytogenetic status was defined by FISH analysis. Standard-risk denoted absence of HiRCAs and +1q. Non-1q HiRCAs included t(4;14), t(14;16), del(17p), and del(1p32).





**Figure S4**. Univariate analysis of PFS in patients with available cytogenetic data.
CI= 95% confidence of interval; EST= estimates; HiR= high-risk; HiRCA= high-risk cytogenetic abnormality; ISS= International Staging System; n= number of patients; NA= not available; OS= overall survival; p= p-value; Pd= pomalidomide and dexamethasone; Rd= lenalidomide and dexamethasone; SR= standard-risk; Vd= bortezomib and dexamethasone; VMP= bortezomib, melphalan and prednisone; VTd= bortezomib, thalidomide and dexamethasone; +1q= gain/amplification of 1q; +1q+HiRCAs= +1q plus ≥ 1 non-1q HiRCAs.
Renal insufficiency was defined as creatinine clearance <60 mL/min, calculated using the CKD-EPI equation.
HiR clinical features include extra-medullary disease, secondary plasma cell leukemia and circulating plasma cells < 5%.
Cytogenetic status was defined by FISH analysis. Standard-risk denoted absence of HiRCAs and +1q. Non-1q HiRCAs included t(4;14), t(14;16), del(17p), and del(1p32).





**Figure S5**. Univariate analysis of TTNT in patients with available cytogenetic data.
CI= 95% confidence of interval; EST= estimates; HiR= high-risk; HiRCA= high-risk cytogenetic abnormality; ISS= International Staging System; n= number of patients; NA= not available; OS= overall survival; p= p-value; Pd= pomalidomide and dexamethasone; Rd= lenalidomide and dexamethasone; SR= standard-risk; Vd= bortezomib and dexamethasone; VMP= bortezomib, melphalan and prednisone; VTd= bortezomib, thalidomide and dexamethasone; +1q= gain/amplification of 1q; +1q+HiRCAs= +1q plus ≥ 1 non-1q HiRCAs.
Renal insufficiency was defined as creatinine clearance <60 mL/min, calculated using the CKD-EPI equation.
HiR clinical features include extra-medullary disease, secondary plasma cell leukemia and circulating plasma cells < 5%.
Cytogenetic status was defined by FISH analysis. Standard-risk denoted absence of HiRCAs and +1q. Non-1q HiRCAs included t(4;14), t(14;16), del(17p), and del(1p32).





**Figure S6**. Univariate analysis of OS in patients with available cytogenetic data.
CI= 95% confidence of interval; EST= estimates; HiR= high-risk; HiRCA= high-risk cytogenetic abnormality; ISS= International Staging System; n= number of patients; NA= not available; OS= overall survival; p= p-value; Pd= pomalidomide and dexamethasone; Rd= lenalidomide and dexamethasone; SR= standard-risk; Vd= bortezomib and dexamethasone; VMP= bortezomib, melphalan and prednisone; VTd= bortezomib, thalidomide and dexamethasone; +1q= gain/amplification of 1q; +1q+HiRCAs= +1q plus ≥ 1 non-1q HiRCAs.
Renal insufficiency was defined as creatinine clearance <60 mL/min, calculated using the CKD-EPI equation.
HiR clinical features include extra-medullary disease, secondary plasma cell leukemia and circulating plasma cells < 5%.
Cytogenetic status was defined by FISH analysis. Standard-risk denoted absence of HiRCAs and +1q. Non-1q HiRCAs included t(4;14), t(14;16), del(17p), and del(1p32).


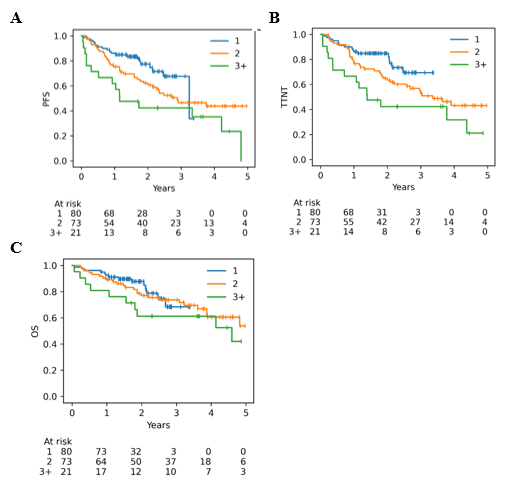


**Figure S7.** Survival outcomes of the overall cohort stratified by line of treament. **A.** Progression-Free Survival. **B.** Time to next treatment. **C.** Overall Survival.


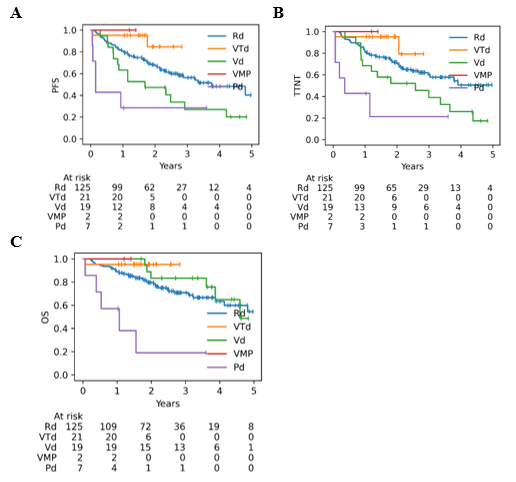


**Figure S8.** Survival outcomes of the overall cohort stratified by treatment scheme. **A.** Progression-Free Survival. **B.** Time to next treatment. **C.** Overall Survival.


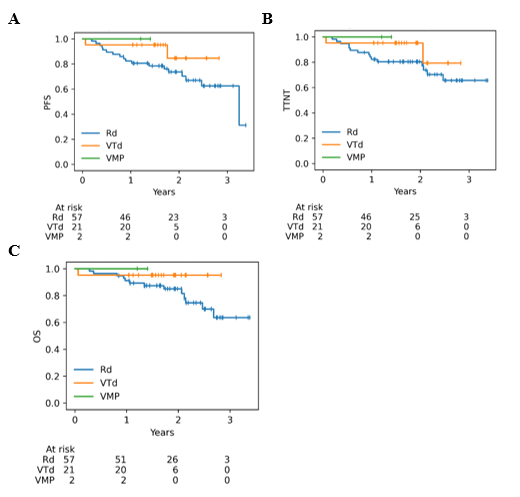


**Figure S9.** Survival outcomes of NDMM patients (i.e. first-line treatment) stratified by treatment scheme. **A.** Progression-Free Survival. **B.** Time to next treatment. **C.** Overall Survival.


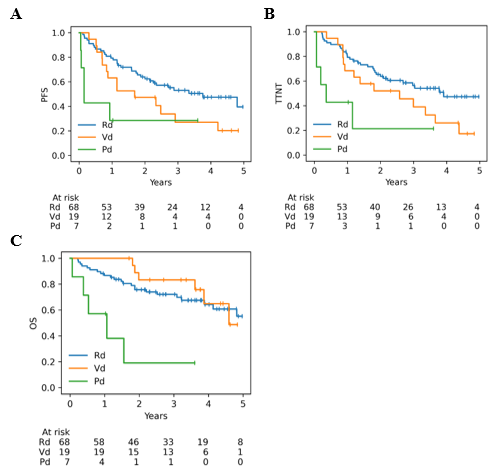


**Figure S10.** Survival outcomes of RRMM patients (i.e. ≥2 lines of treatment) stratified by treatment scheme. **A.** Progression-Free Survival. **B.** Time to next treatment. **C.** Overall Survival.


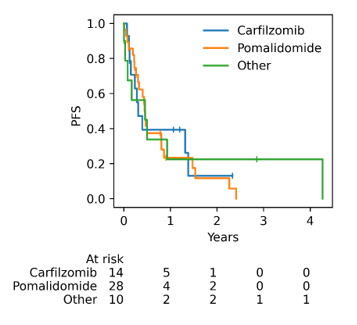


**Figure S11.** Progression-Free Survival of patients relapsed/refractory to daratumumab-based treatments stratified by salvage treatment scheme.


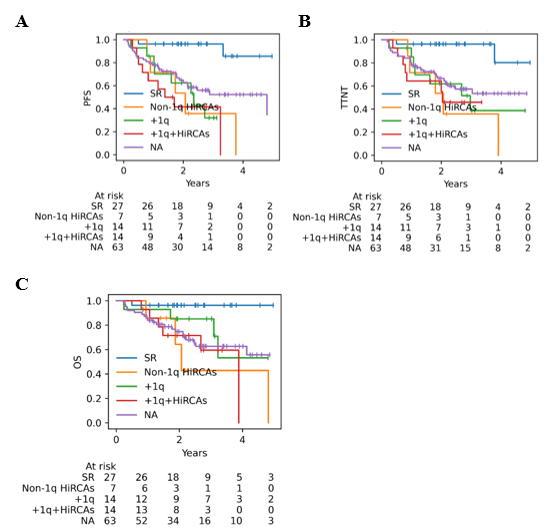


**Figure S12.** Survival outcomes of the DaraRd subgroup stratified by cytogenetic status. **A.** Progression-Free Survival. **B.** Time to next treatment. **C.** Overall Survival.


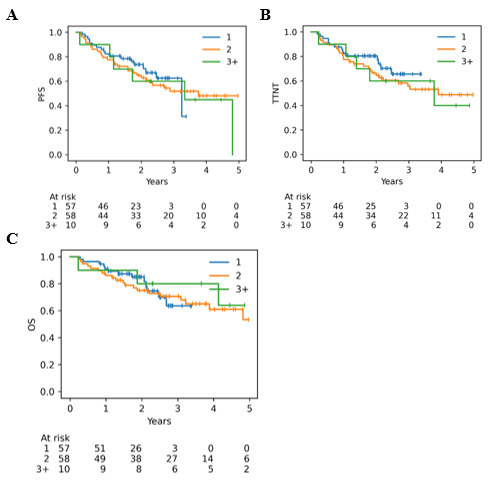


**Figure S13.** Survival outcomes of the DaraRd subgroup stratified by line of treatment. **A.** Progression-Free Survival. **B.** Time to next treatment. **C.** Overall Survival.
